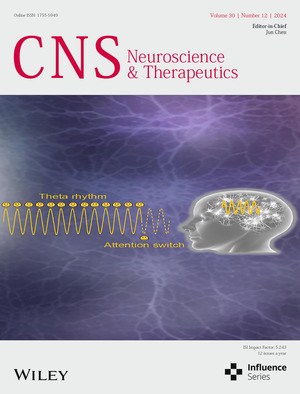# Front Cover

**DOI:** 10.1111/cns.70212

**Published:** 2024-12-31

## Abstract

Cover image: The cover image is based on the article *Theta Rhythm-Based Attention Switch Training Effectively Modified Negative Attentional Bias* by Yifeng Wang et al., https://doi.org/10.1111/cns.70157.